# Impact of Air Pollution on the Composition and Diversity of Human Gut Microbiota in General and Vulnerable Populations: A Systematic Review

**DOI:** 10.3390/toxics10100579

**Published:** 2022-09-30

**Authors:** Simone Filardo, Marisa Di Pietro, Carmela Protano, Arianna Antonucci, Matteo Vitali, Rosa Sessa

**Affiliations:** Department of Public Health and Infectious Diseases, University of Rome “Sapienza”, 00185 Rome, Italy

**Keywords:** air pollution, human gut microbiota, systematic review, dysbiosis, vulnerable population

## Abstract

Recently, growing attention has focused on the impact of air pollution on gut microbiota as a possible mechanism by which air pollutant exposure increased the risk for chronic diseases, as evidenced by in vivo studies demonstrating important exposure-induced alterations in the diversity and relative abundance of gut bacterial taxa. This systematic review provides updated state-of-art findings of studies examining the impact of air pollution on the human gut microbiota. Databases PubMed, Scopus, and Web of Science were searched with the following strategy: “air poll*” AND “gut micro*” OR “intestinal micro*”; moreover, a total of 10 studies were included. Overall, there is the evidence that short-term and long-term exposure to air pollutants have the potential to alter the composition and diversity of gut microbiota; some studies also correlated air pollution exposure to adverse health effects (impaired fasting glucose, adverse pregnancy outcomes, and asthma attacks) via alterations in the composition and/or function of the gut microbiota. However, the evidence on this topic is still scarce, and large cohort studies are needed globally.

## 1. Introduction

Air pollution represents one of the most important environmental threats to public health globally, and it is responsible for an increased risk of morbidity and mortality due to cardiopulmonary and neoplastic diseases [1,2]. Epidemiological studies have shown an association between exposure to air pollutants and an increased incidence of asthma and chronic obstructive pulmonary disease [3,4,5]; their exposure has also been associated with the development of lung cancer [6,7,8]. Lastly, exposure to air pollution has been linked with an increased risk of stroke and ischemic heart disease [9,10].

According to the World Health Organization, around 90% of individuals globally, living in urban areas, are exposed to air containing high levels of pollutants, and about 4.2 million people die prematurely from outdoor air pollution each year [2,11]. In urban areas, rapid economic development and urbanization have concurrently boosted energy consumption and pollutant emissions, which has led to increasingly serious environmental pollution [12]. The air pollutants include gaseous components (carbon oxides, nitrogen oxides, sulphur oxides, and ozone), volatile organic compounds (e.g., hydrocarbons also halogenated), and fine and ultrafine particulate matter (PM) that may contain metals, semi-metals, and persistent organic pollutants. All these contaminants may determine deleterious effects on human health [13].

Evidence has been provided on air pollutant’s ability to directly irritate airway epithelium and cause oxidative stress and inflammation, underlying the development or progression of respiratory diseases, including lung cancer [14,15]. In addition, air pollutants may reach the gastrointestinal tract through the muco-ciliary clearance of inhaled pollutants or the intake of contaminated food and water [14]. Once in the gastrointestinal tract, air pollutants may interact with the intestinal epithelium, promoting the production of reactive oxygen species and pro-inflammatory oxidative lipids and contributing to the development of intestinal diseases [16,17]. In support of this hypothesis, recent studies have associated air pollution with intestinal diseases [13], including colorectal cancer [18] and inflammatory bowel diseases [19].

More recently, growing attention has focused on the impact of air pollution on gut microbiota as a possible mechanism by which air pollutant exposure increases the risk for chronic diseases. In this regard, particularly interesting are in-vivo studies demonstrating important exposure-induced alterations in the diversity and relative abundance of gut bacterial taxa [20,21,22].

Intestinal microbiota is more complex than other microbiota niches [23,24,25], for instance, the genital microbiota [26,27], and accounts for approximately 10^13^ bacteria; the most dominant bacterial phyla are *Firmicutes* (60–80%) and *Bacteroidetes* (20–40%), while *Proteobacteria*, *Actinobacteria*, *Fusobacteria*, and *Verrucomicrobia* are in the minority. Typically, obligate anaerobes (e.g., *Bacteroides*, *Clostridium*, *Fusobacterium*, and *Bifidobacterium*) prevail over facultative anaerobes (e.g., *Lactobacillus*, *Escherichia*, and *Enterococcus*) and the balance of the intestinal microbiota is essential for the integrity of the intestinal epithelial barrier and its functions as well as for the protection against pathogenic challenges. On the contrary, a disbalance of the gut microbiota composition, namely dysbiosis, has been associated with several chronic conditions, such as inflammatory bowel disease [28], cancer [29], and metabolic disorders [30].

The intestinal microbiota composition is highly variable among individuals; specifically, the abundance of specific bacteria varies in relation to several factors, including age, diet, the administration of antibiotics, and, as recently highlighted, environmental pollution exposure [31].

This systematic review provides updated state-of-art findings of studies examining the impact of air pollution on the human gut microbiota. Potential mechanisms by which air pollution can cause adverse health effects via the alteration of human microbiota are also described.

## 2. Materials and Methods

### 2.1. Selection Protocol and Search Strategy

This systematic review was registered in the International Prospective Register of Systematic Reviews, registration number: CRD42022328390. The protocol was written according to the Preferred Reporting Items for Systematic Review and Meta-Analysis (PRISMA) statement [32]. Zotero citation management software (RRID: SCR_013784) was used to identify any duplicates and to manage and screen the selected literature records.

The research study was performed between 23rd and 31st May 2022 in databases PubMed, Web of Science, and Scopus. We restricted the publication language to English. Relevant studies were identified using the following keyword and Boolean operator combinations: “air poll*” AND “gut micro*” OR “intestinal micro*”. Truncation filters (*) were used to represent any combination of letters. Three independent reviewers (SF, MDP, and CP) performed the search, reading the titles and abstracts of the articles identified by the search strategy. During the multi-step exclusion process, disagreements on the studies were discussed until reaching a consensus. The process was supervised by other investigators (MV and RS). The PRISMA flow chart diagram was used for summarizing the selection steps for the present systematic review.

### 2.2. Study Selection and Eligibility Criteria

Three (SF, MDP, and CP) of the co-authors independently performed study selection using the Population, Intervention, Comparison, Outcomes, and Study (PICOS) criteria for inclusion and exclusion criteria. We included all studies meeting the following criteria: (1) all genders and all age individuals; (2) exposure to ambient air pollution; (3) gut dysbiosis outcome. Exclusion criteria were as follows: (1) exposure to indoor air pollution; (2) respiratory outcomes and skin microbiota dysbiosis. Animal studies, reviews, systematic reviews, meta-analysis, editorials, commentaries, case reports, case series, semi-experimental and experimental studies, proceedings, individual contributions (e.g., conference speeches), and purely descriptive studies published in scientific conferences without any quantitative or qualitative findings were excluded from the review.

### 2.3. Data Extraction Process and Quality Assessment

Three authors (SF, MDP, and CP) independently extracted the following information from each included study: first author’s name, publication year, study region, study period, study population, sample size, study design, parameters of air pollution, duration of exposure, type of exposure, exposure assessment methods, and confounding factors. Differences in the composition and diversity of gut microbiota and any measure of association to air pollutants were reported as main results. Two different reviewers (MV and RS) assessed the methodological quality of the selected studies with the Newcastle–Ottawa Scale (NOS) rating tool, adapted for evaluating case–control, cross-sectional, and cohort studies [33]. The NOS is divided in eight categories evaluating three different quality aspects: selection, comparability, and outcome; scores range from 0 to 9, and the quality of a study was high if the NOS score was 7 to 9, intermediate if the NOS score was 4 to 6, and low if it was 0 to 3 [34].

Studies were also assessed according to the “Strengthening The Organization and Reporting of Microbiome Studies” (STORMS) checklist criteria for reporting metagenomic studies [35].

## 3. Results

### Study Selection and Characteristics

**Figure 1:**
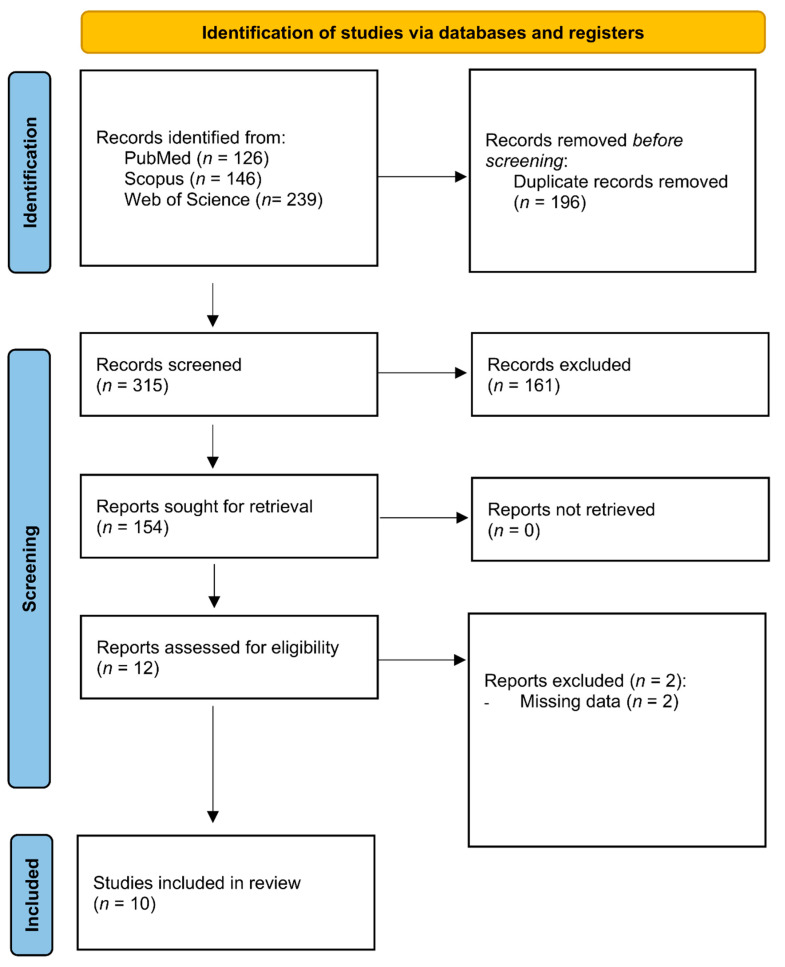
PRISMA flow diagram.

After searching the designated databases, 511 articles were retrieved. A total of 196 articles were excluded after removing duplicates. Out of the remaining 315 papers, 154 were subjected to further screening and evaluated for inclusion in the systematic review after considering inclusion and exclusion criteria. Finally, 12 articles were considered eligible to be included in the systematic review, but 2 articles were excluded for missing data. A total of 10 articles were used for the data extraction [36,37,38,39,40,41,42,43,44,45]. The PRISMA flow diagram in Figure 1 illustrates the search results.

Table 1 and Table 2 described the characteristics of the studies included in the systematic review.

**Table 1:** General characteristics of the studies included in the systematic review.

Authors Study Period Country/Location	Study Population	Sample Size	Age Range (Years)	Air Pollutants	Duration	Exposure Assessment	Main Results
Alderete et al., 2014–2016 USA [45]	Overweight and obese adolescents	43 (42% female)	17–19	Traffic related air-pollution (TRAP), measured as modeled NOx exposure	long-term (12 months)	California Line Source Dispersion Model	Decreased *Bacteroidaceae* (r = − 0.48; *p* = 0.001) and increased *Coriobacteriaceae* (r = 0.48; *p* < 0.001) were associated with TRAP exposure and explained 24% and 29% of its correlation with fasting glucose levels (r = 0.45; *p* = 0.04).
Du et al., 2019 China [40]	Healthy subjects	2507 (63% female)	40–63	Air-pollution related to increasing levels of urbanization	long-term (>6 months)	Degree of urbanization: rural and urban area (MR, mountainous rural; MU, mountainous urban; PR, plain rural; PU, plain urban)	Lower diversity of gut microbiota with higher levels of urbanization, characterized by gradually decreasing Firmicutes and Actinobacteria and increasing Proteobacteria, as urbanization deepened (MR -> MU -> PR -> PU).
Fouladi et al., 2014–2017 USA [39]	Overweight young adults	101 (42% female)	17–22	NO_2_, PM_10_, PM_2.5_, O_3,_ total NOx	long-term (12 months)	U.S. Environmental Protection Agency’s Air Quality System; California Line Source Dispersion Model for NOx levels	Decreased Shannon’s diversity index, with higher relative abundance of *Bacteroides caecimuris*, as well as different metabolic pathways, such as L-ornithine de novo biosynthesis. Pantothenate and coenzyme A biosynthesis I were correlated to O_3_ exposure for 24 h. Moreover, NO_2_ exposure correlated to fewer taxa. Interestingly, O_3_ exposure was responsible for up to 11.2% variability of the gut bacterial composition.
Gan et al., 2017–2018 China [38]	Pregnant women	916	20–44	SO_2_, NO_2_, O_3_, PM_10,_ PM_2.5_	long-term (3, 6, 9 months)	National Air Quality Monitoring Stations	An altered gut microbiota was significantly associated with air pollution exposure during pregnancy, increasing the risk of various APOs (Adverse Pregnancy Outcomes, such as pre-term birth, post-term birth, low birth weight, macrosomia fetus, birth defect, pathological caesarean section, and post-partum haemorrhage) by 1.07–1.36-fold (*p* < 0.05). *Eggerthella* spp., *Phascolarctobacterium* spp. and *Clostridium* spp. seemed to be partially responsible of the effects of air pollutants (PM_2.5_, PM_10_, O_3_, NO_2_, and SO_2_) on APOs. *Micrococcaceae* was responsible for 11.39%, 64.90% and 54.80% of the correlation between SO_2_, PM_2.5_, PM_10,_ and POTB, respectively, whereas *Rothia* spp. Was responsible for 11.97%, 67.80%, and 54.50%, respectively. *Parabacteroides* spp. were instead responsible for 53.0% of the correlation between PM_2.5_ and pre-term birth.
Li et al., 2018–2019 China (Danliu community of Jinan, with no factories within at least 5 km) [37]	Healthy elderly subjects	76 (51% female)	55–74	PM_2.5_	3 days	Real-time personal exposure measured via MicroPEM sensors	PM_2.5_ exposure was significantly associated with more than 20 gut microbial species. More than 600 metabolites (Human Metabolome Database) were identified via untargeted metabolomic analysis, and 253 of them showed a statistically significant (FDRB-H < 0.05) association with PM_2.5_ exposure. Four different tryptophan metabolites were significantly associated with PM_2.5_ exposure.
Liu et al., 2015–2016 China (14 districts of Guangdong Province) [36]	Adults	6627 (55% female)	18 and older (average mean = 52 years old)	PM_2.5_, SO_2_, NO_2_, CO	long-time (two years)	Spatiotemporal land-use regression model	Impaired Fasting Glucose (IFG) and type 2 diabetes were at a higher risk in individuals exposed to PM_2.5_ and PM_1_ long term. Alterations in the gut microbiota may partially be responsible for the effects of PM. Firmicutes, Proteobacteria, and Verrucomicrobia were negatively associated with the levels of PM and the risk of diabetes. Some *Firmicutes* spp., such as *Lachnospiraceae* and *Clostridiaceae*, were responsible for more than 10% of PMS’ effects on type 2 diabetes.
Vari et al., 2015 Finland (rural and urban area of Lahti city) [44]	Elderly people	62 (48% female)	65–79	PAHs	28 days	Passive sampling device placed in rural area (*n* = 30) and urban area (*n* = 32) of Lahti	Location of households close to broad-leaved and mixed forests might favour the functional potential of human gut microbiota, increasing orthologues for peroxisome proliferator-activated receptor (PPAR) pathway. These households had lower PAH levels, suggesting the capture of gaseous PAHs by broad-leaved trees. In fact, forests reduce the negative health risks induced by PAH pollution and may balance the commensal microbiota.
Yi et al., 2017–2019 China (Anhui Mental Health Center) [43]	Subjects with schizophrenia	248 (63% female)	18 and older (average mean = 37 years old)	PM_2.5_, PM_10_, O_3_, NO_2_, SO_2_ and CO	long-term (12 months)	Spatially interpolated by Inverse Distance Weighted interpolation algorithm b (individual exposure estimates of air pollutants)	Nitrogen dioxide (NO_2_), carbonic oxide (CO), ozone (O_3_), particulate matter with lower diameter than 10 μm (PM_10_), and fine particulate matter (PM_2.5_) induced 2.68% to 10.77% of the gut microbiome alterations in schizophrenia patients (*p* < 0.05). Network correlation analysis showed the correlation between air pollutants, markers of liver function, and Firmicutes, Actinobacteria, and Proteobacteria.
Zhao et al., 2018–2019 China (Danliu community in Shandong Province) [42]	Elderly subjects	76 (51% female)	60–69	PM_2.5_	3 days	Real-time personal exposure via MicroPEM sensors	Increased risk of higher insulin resistance (IR) index was significantly associated with PM_2.5_ exposure. The gut microbiota (Shuttleworthia) was responsible for 37.83% of PM_2.5_ total effect on sphingolipid metabolism, suggesting that it may contribute to systemic inflammation and altered sphingolipid metabolism via alterations of the gut microbiota.
Zheng et al., 2017 China (Beijing) [41]	11 asthmatic children and 10 healthy children	21 (38% female)	5–12	PM_2.5_, PM_10_, NO_2_, SO_2_, O_3_	5 days	Monitoring station. Air Quality Index (clean day < 100; smog day > 100) in according to Technical Regulation on Ambient Air Quality Index, Ministry of Environmental Protection	Gut microbiota composition varied between clean and smog days amongst all children (PERMANOVA, *p* = 0.03). The gut microbiota of asthmatic children was characterized, in smog days, by a decrease in the levels of *Bifidobacteriaceae*, *Erysipelotrichaceae*, and Clostridium sensu-stricto 1 and an increase in *Streptococcaceae*, *Porphyromonadaceae*, *Rikenellaceae*, *Bacteroidales* S24-7 group, and *Bacteroides* (Wilcoxon test, *p* < 0.05). By contrast, healthy children experienced a decrease in *Fusicatenibacter* and an increase in *Rikenellaceae* and *Terrisporobacter* (Wilcoxon test, *p* < 0.05). The abundance of some bacteria belonging to Firmicutes was negatively correlated with PM_2.5_, PM_10_, NO_2_, and SO_2_ (multiple linear regression, *p* < 0.05).

NOx, nitrogen oxides; NO_2_, nitrogen dioxide; PM, particulate matter; O_3,_ ozone; SO_2_,sulfur dioxide; CO, carbon monoxide; PAHs, polycyclic aromatic hydrocarbons.

The included studies (nine cross-sectional and one cohort study) were published from 2018 to 2022 [36,37,38,39,40,41,42,43,44,45] (Table 2). Among the ten studies, two of them were conducted in America [39,45], seven in China [36,37,38,40,41,42,43], and one in Europe (Finland) [44], and the sample size was very variable, ranging from 21 to 6627 subjects.

The studies included healthy individuals as well as vulnerable populations such as obese adolescents, asthmatic children, schizophrenic patients, and pregnant women with adverse pregnancy outcomes. Both male and female were enrolled in all studies, with the exception of one study that included females only [38]. Children, adolescents, young adults, adults, and older people were included, with ages ranging from 5 to over 70 years. 

Half of the air pollution studies (*n* = 5; 50%) examined multiple air pollutants: PM_10_ (*n* = 4; 40%), PM_2.5_ (*n* = 7; 70%), O_3_ (*n* = 4; 40%), NOx (*n* = 6; 60%), SO_2_ (*n* = 4; 40%), and CO (*n* = 2; 20%) [36,38,39,41,43]. Lastly, one study analysed polycyclic aromatic hydrocarbons (PAHs) [44] and one focused on the levels of urbanization [40].

Regarding exposure time, short-term exposure (from 2 to 5 days) to air pollutants was considered in four studies [37,41,42], whereas long-term exposure (28 days to 2 years) was investigated in six studies [36,38,39,40,44,45]. Moreover, the methods of exposure assessments varied among studies: daily air quality index scores assigned using the monitoring data of China National Environmental Monitoring Centre [38], levels of urbanization [40], ambient monitoring station [41], real-time personal exposure [37,42], passive sampling devices [44], and other various modelled estimates (*n* = 3) [36,43,45] were used (Table 1).

As for the STORMS metagenomic checklist, all included studies provided a partial description of methods for sample collection, storage, and DNA isolation. Sequencing methods were reported by almost all included studies. 16S rDNA gene sequencing was performed in nine studies by using Illumina Hiseq or Miseq platforms, whereas whole-genome shotgun sequencing was conducted in one study [39] via the Illumina Hiseq platform. The region of amplification of the 16S rDNA gene varied among studies, such as V4 (*n* = 4), V3–V4 (*n* = 2), and V5 (*n* = 1) (Table 2). Two studies mentioned that they used three different regions (V4, V3–4, and V4–5), but it was unclear how these analyses were integrated into bacteria characterization [37,42]. Moreover, the primers used for the amplification varied across studies: three studies used 515F/806R primers, and two used 341F/805R and 338F/806R, respectively (Table 2).

The majority of the studies (*n* = 8) reported quantitative measures of microbial diversity and richness within bacterial community via different alpha-diversity indices (e.g., Chao1’s, Shannon’s, and Simpson’s indices). Two studies reported a β-diversity measure [41,42] to evaluate differences in microbial abundances between multiple samples. Lastly, three studies performed linear discriminant analyses for identifying potential markers of dysbiosis associated with air pollution exposure [38,40,41]. Functional gene pathways were investigated in two studies [39,41]. Concerning the study quality assessment, the median NOS score was 6 (interquartile range 1.75), hence indicating an intermediate average level. Specifically, *n* = 4 studies were of high quality (NOS equal to 7), and *n* = 6 studies were of intermediate quality (score of 5 to 6). Table S1 shows the results of the scoring method applied to each study included in the review, with reference to publication year.

Overall, a significant correlation between exposure to specific air pollutant and alteration in human gut microbiota was found, and it did not depend on duration of exposure (short- and long-term exposure). A significant association was found in healthy individuals as well as vulnerable populations.

**Table 2:** Study design, 16S rDNA sequencing regions, primers, and sequencing platform adopted by the included studies.

Author	Year	Study Design	16S rDNA Sequencing Region	Primers	Sequencing Platform
Alderete et al. [45]	2018	Cross-sectional study	V4	515F; 806R	Illumina Miseq v3
Liu et al. [36]	2019	Cross-sectional study	V4	Not reported	Not reported
Fouladi et al. [39]	2020	Cross-sectional study	WGS	--	Illumina HiSeq 4000
Zheng et al. [41]	2020	Cross-sectional study	V4	515F; 806R	Illumina HiSeq 2500
Du et al. [40]	2021	Cross-sectional study	V3–4	341F; 805R	Illumina Miseq
Yi et al. [43]	2021	Cross-sectional study	V3–4	338F; 806R	Illumina MiSeq 300
Vari et al. [44]	2021	Cross-sectional study	V4	515F; 806R	Illumina MiSeq
Gan et al. [38]	2022	Cohort study	V5	515F; 807R	Illumina Miseq
Li et al. [37]	2022	Cross-sectional study	V4; V3–4; V4–5	Not reported	Illumina HiSeq 2500
Zhao et al. [42]	2022	Cross-sectional study	V4; V3–4; V4–5	515F; 806R	Illumina HiSeq 2500

WGS, Whole Genome Sequencing.

## 4. Discussion

This systematic review assembled the currently available evidence on the impact of air pollution on human gut microbiota. All 10 revised studies evidenced that the exposure to air pollutants has the potential to alter the composition of gut microbiota [36,37,38,39,40,41,42,43,44,45]; some of them also correlated air pollution exposure to negative effects on human health via alterations in composition and/or function of the gut microbiota [36,38,41,43,45], and only three studies characterized some mechanisms by which air pollutants may have adverse health effects via the dysregulation of gut microbiota [37,39,42].

Concerning the effects of air pollution on the composition of gut microbiota, long-term (from 6 months up to 24 months) exposure to O_3_, NO_2_, SO_2_ PM_10_, PM_2.5_, and PM_1_, as well as to traffic-related air-pollution (TRAP), has been shown to alter richness and diversity of human gut microbiota independently from age and gender of the study’s populations. For example, higher O_3_ exposure was associated with a higher abundance of bacterial species belonging to the *Bacteroidaceae* family [39]. Higher NO_2_ levels were associated with a higher abundance of *Coriobacteriaceae* [39], and freeway TRAP exposure was correlated to decreased *Bacteroidaceae* and increased *Coriobacteriaceae* [45]. Lastly, short-time exposure to air pollutants (PM_2.5_ for 3 days) was also demonstrated to induce alterations in gut microbiota, characterized by an increased abundance of a varied mix of both beneficial and harmful bacteria [37].

Several interesting evidence showed that the interplay between gut microbiota and air pollution also possessed negative effects on human health. Shifts in the relative abundance of *Eggerthella*, *Phascolarctobacterium*, and *Clostridium* have been described to partially mediate the effects of multiple air pollutants (PM_2.5_, PM_10_, O_3_, NO_2_, and SO_2_) on adverse pregnancy outcomes [38]. In young adults, TRAP-mediated decreased abundances of *Bacteroidaceae* and increased abundances of *Coriobacteriaceae* were correlated with impaired fasting glucose, a known risk factor for type 2 diabetes [45]. Lastly, in adults, a decreased abundance of *Firmicutes*, *Proteobacteria*, and *Verrucomicrobia*, induced by PM_2.5_ and PM_1_ exposure, increased the risk of impaired fasting glucose and type 2 diabetes [36].

New insights into the mechanisms by which air pollutants can cause adverse health effects via the dysregulation of gut microbiota are of pathological importance. On this regard, functional microbial gene pathways related to cell growth and insulin release (L-ornithine de novo biosynthesis, pantothenate, and coenzyme A biosynthesis I) were found to be associated with O_3_ exposure. This supported the etiological role of gut microbiota in the association between air pollution and metabolic disorders, such as type 2 diabetes [39]. Zheng et al. suggested that the smog-dependant shift of gut microbiota with reduced abundances of *Clostridia* may trigger asthma attacks [41]. As a matter of fact, *Clostridia* can synthesize propionic acid or butyrates, which are known to stimulate the production of regulatory T cells that influence airway health indirectly through the gut–lung axis [46]. Interestingly, air pollution may simultaneously impact gut and lung health because, over the years, it has been proved that gut and lungs can communicate and influence each other, and they are connected via blood circulation and lymphatic system [47,48]. As a result, immune cells, cytokines, chemokines, and microbial metabolites can travel from an organ to the other, affecting its health. In this scenario, it is likely that lung inflammation and shifts in lung microbiota, following the exposure to air pollution, may lead to gut dysbiosis and, hence, to the onset or progression of intestinal pathologies.

Different potential mechanisms of damage were also described for other air pollutants; PM_2.5_ may affect sphingolipid metabolism, which is partially mediated by the decreased abundance of *Shuttleworthia*, contributing to insulin resistance and, hence, type 2 diabetes [42]. Another study suggested that PM_2.5_ exposure may result in changes in tryptophan metabolism, which is associated with the imbalance in the gut microbiota, thereby activating the gut–brain axis through the production of central neurotransmitters such as serotonin [36]. Specifically, tryptophan, a precursor of serotonin, is stored in limited quantities in the brain, and continuous supplementation from the digestive system is required for brain development and function [49].

The main strengths of the reviewed studies are the characterization of gut microbiota via high-throughput sequencing technologies (16S rDNA sequencing and shotgun metagenomics), as well as the accurate analysis of outdoor air pollutants. Moreover, the application of stringent inclusion criteria allowed greatly diminishing the impact of confounding bias related to the selection of the study population. In this regards, potentially important confounder factors known to be associated with the exposure to air pollutants and/or with the gut microbiota (for example, smoke or therapy with antibiotics) were considered.

Despite all the compelling evidence highlighted in this systematic review, a long road is still ahead of us before reaching clinically relevant conclusions due to the small number of papers (*n* = 10) and their weaknesses. 

First, a high heterogeneity of sample size and type, as well as of exposure time, methods of exposure assessment, and metagenomic analysis, was observed. In particular, sample sizes were very variable among studies; notably, two studies [41,45] enrolled a low number of individuals (*n* = 21 and 42, respectively), and their results should be considered with caution. Moreover, the study populations included different categories of individuals, including pregnant women [38], children [41], young adults [39,45], adults [36,40,43], and older adults [37,42,44], with ages ranging from 5 to over 70 years. In this regard, it is well known that gut microbiota composition and diversity vary from infants to the elderly [50]. Of note, studies also included vulnerable populations, such as obese adolescents, asthmatic children, elderly, and pregnant women, which might be more susceptible to the negative effects of air pollution exposure. Concerning exposure time and assessment, these also varied among studies; time of exposure ranged from 3 days [37,42] to two years [36], which may lead to different changes in gut microbial composition. Daily air quality index scores assigned using the monitoring data of the China National Environmental Monitoring Centre [38], levels of urbanization [40], ambient monitoring station [41], real-time personal exposure [37,42], passive sampling devices [44], and other various modelled estimates [36,39,43,45] were used. It is well-known that the usage of different approaches in the assessment of air pollution exposure results in different accuracies and precision of estimates [51]. The most accurate method for assessing pollutant exposure seems to be personal monitoring, particularly biological monitoring; however, when the number of enrolled subjects is high, determining air pollutants levels can be suitable by using a variety of exposure assessment models [51]. This issue should be carefully considered for future research in the field. Lastly, it is very challenging to compare the biodiversity and the composition of the gut microbiota between different studies and patient groups due to different primers and hypervariable regions of 16S rDNA chosen for sequencing, alongside the numerous and diverse statistical measures used for bioinformatic analyses.

Second, almost all reviewed studies were observational, limiting the ability to provide a proof of causality. 

Third, most of the examined studies enrolled mostly Chinese populations with distinct eating habits known to have impacts on the composition and diversity of the human gut microbiota [52]. The considerable disease burden attributable to air pollution following rapid economic development might explain the high number of studies in China [53].

Another relevant issue emerging from this review is that the overall risk of bias was partially considered in individual studies. For example, important information such as indoor activity time (home, school, and university) and indoor air pollution exposure, known to influence the composition of gut microbiota [54], was not considered. Moreover, the interplay between the different air pollutants and gut microbiota in the onset and development of chronic diseases has not been investigated. Similarly, nutrition and diet effects cannot be fully controlled, considering the long-term exposure to air pollution in most studies (>50%).

## 5. Conclusions

Overall, the exposure to air pollutants may be able to induce significant alterations in the composition of the gut microbiota. In addition, potential mechanisms by which air pollution may contribute to adverse health effects, such as impaired fasting glucose, adverse pregnancy outcomes, and asthma attacks via alterations in composition and/or function of the gut microbiota, were reported. However, the current evidence is weak due to the small number of papers, observational design of the included studies, as well as the high heterogeneity of the methods used for human microbiome and air pollution analyses. As a result, it will be of great interest to reach a consensus on the several parameters involved in designing metagenomic studies related to air pollution. Surely, an improved standardization of the methodological approach, from sample collection and storage to DNA extraction, genome amplification, and sequencing methods, is needed to obtain increased data accuracy, reproducibility, and comparability of results. In the future, large cohort studies worldwide based on simultaneous stool and respiratory sampling with the application of STORMS checklist for metagenomic analysis [35], as well as biological monitoring, will allow gaining deeper insights into the impact of air pollution on the growing prevalence of chronic diseases, although this study type is expensive and time consuming.

## Data Availability

Not applicable.

## Supplementary Materials

The following supporting information can be downloaded at: https://www.mdpi.com/article/10.3390/toxics10100579/s1, Table S1: NOS score of the studies included in the systematic review.
