# Supplementary material for: Impact of Air Pollution on the Composition and Diversity of Human Gut Microbiota in General and Vulnerable Populations: A Systematic Review
Source: Toxics. 2022 Sep 30;10(10):579. doi: 10.3390/toxics10100579 (PMC9607944; doi:10.3390/toxics10100579)
Supplement: Supplementary file 1 [file toxics-10-00579-s001.zip › toxics-1871374-supplementary.pdf]

toxics

MDPI

Supplementary Materials: Impact of Air Pollution on the Composition and Diversity of Human Gut Microbiota in General and Vulnerable Populations: A Systematic Review

Simone Filardo, Marisa Di Pietro, Carmela Protano, Arianna Antonucci, Matteo Vitali and Rosa Sessa

Table S1. NOS score of the studies included in the systematic review.

Author	Year	NOS Score
Alderete *et al.* [34]	2018	5
Liu *et al.* [25]	2019	7
Fouladi *et al.* [28]	2020	6
Zheng *et al.* [30]	2020	6
Vari *et al.* [33]	2021	6
Du *et al.* [29]	2021	5
Yi *et al.* [32]	2021	7
Gan *et al.* [27]	2022	5
Li *et al.* [26]	2022	7
Zhao *et al.* [31]	2022	7
